# Two New Picoline-Derived Meroterpenoids with Anti-Acetylcholinesterase Activity from Ascidian-Derived Fungus *Amphichorda felina*

**DOI:** 10.3390/molecules27165076

**Published:** 2022-08-10

**Authors:** Minghua Jiang, Heng Guo, Qilin Wu, Siwen Yuan, Lan Liu

**Affiliations:** 1 School of Marine Sciences, Sun Yat-sen University, Zhuhai 519082, China; 2 Southern Marine Science and Engineering Guangdong Laboratory (Zhuhai), Zhuhai 519000, China; 3 Pearl River Estuary Marine Ecosystem Research Station, Ministry of Education, Zhuhai 519082, China

**Keywords:** fungal meroterpenoid, picoline, *Amphichorda felina*, anti-acetylcholinesterase activity

## Abstract

Amphichoterpenoids D (**1**) and E (**2**), two new picoline-derived meroterpenoids with a rare 6/6/6 tricyclic pyrano[3,2-c]pyridinyl-*γ*-pyranone scaffold, were isolated from the ascidian-derived fungus *Amphichorda felina* SYSU-MS7908. Their structures, including the absolute configurations, were established by extensive spectroscopic methods (1D and 2D NMR and high-resolution mass spectrometry) and ECD calculations. Compounds **1** and **2** showed anti-acetylcholinesterase (anti-AChE) activities with IC_50_ values of 12.5 μM and 11.6 μM, respectively. The binding interactions between **1**, **2**, and AChE were investigated using molecular docking analyses.

## 1. Introduction

Meroterpenoids are a class of hybrid secondary metabolites widely distributed in nature, partially derived from a mixed terpenoid biosynthetic pathway [1,2,3,4]. Fungi are a promising source of meroterpenoids with chemical structural diversity and potent bioactivities [1,2,4]. Significantly, some fungal meroterpenes have been used as clinical drugs or promising leads, e.g., immunosuppressant drugs: mycophenolic acid [5], antimicrobial and anti-angiogenesis agents: fumagillin [6,7], clinical anticancer drugs: antroquinonol and 4-acetyl antroquinonol B [8], anti-inflammatory berkeleyacetal C [9] and anti-acetylcholinesterase (anti-AChE): territrem B [10].

**Figure 1:**
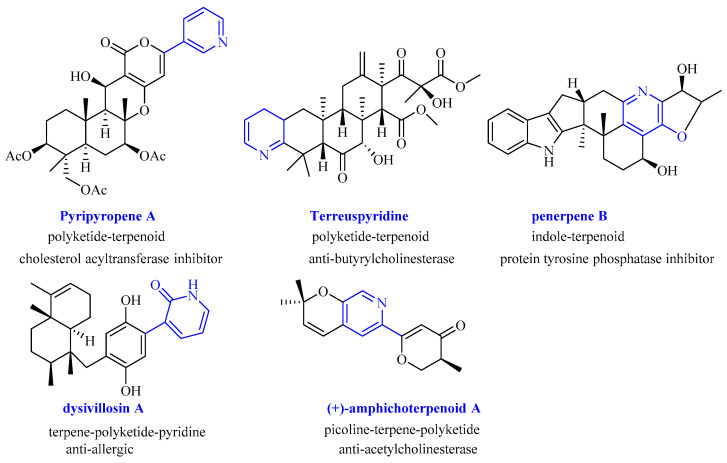
Chemical structure and bioactivities of the representative meroterpenoids with pyridine unit.

Owing to their difference in nonterpenoid starting units, fungal meroterpenoids can be divided into the following four classes: polyketide–terpenoids, indole–terpenoids, shikimate–terpenoids, and miscellaneous meroterpenoids [1,3]. Meroterpenoids possessing pyridine units are a particular class of rare discovered natural products [11,12,13,14,15,16]. Pyridine is a crucial active functional scaffold for many drugs in medicinal chemistry [17]. Thus, these molecules always display rich structural diversity and broad bioactivities (Figure 1), such as cholesterol acyltransferase inhibitor pyripyropenes [11], anti-butyrylcholinesterase terreuspyridine [14], protein tyrosine phosphatase inhibitor penerpene B [13], anti-allergic dysivillosins [15], and anti-AChE amphichoterpenoids [12], which have attracted widespread attention from chemists and biologists to explore their structural diversity, biosynthesis, and bioactivities [11,18,19,20].

**Figure 2:**
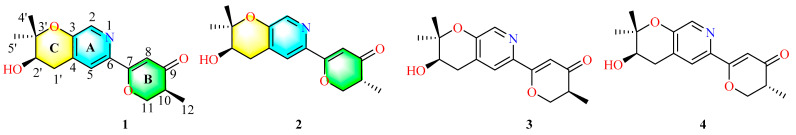
Chemical structures of compounds **1**–**4**.

Marine fungi have been widely recognized as the essential source of bioactive natural products [21,22,23,24,25]. Our research group has focused on discovering secondary metabolites from ascidian-derived fungi [26,27,28,29]. Recently, we have reported anti-AChE meroterpenoids (amphichoterpenoids) [12], antiplatelet and antithrombotic cyclodepsipeptide [30], and anti-inflammatory polyketones [31] from the ascidian-derived fungus *Amphichorda felina* SYSU-MS7908. Amphichoterpenoids are the first example of picoline-derived meroterpenoids featuring a 6/6/6 tricyclic pyrano[3,2-c]pyridinyl-*γ*-pyranone skeleton, with the picoline as the nonterpenoid starting moiety [12]. In order to obtain more of this type of molecules, a chemical investigation of the remaining metabolic components of this fungus strain led to the identification of two new picoline-derived meroterpenoids, amphichoterpenoids D and E, (**1** and **2**) (Figure 2). Herein, the details of the isolation, structural elucidation, anti-AChE activity, and molecular docking studies of compounds **1** and **2** are reported.

## 2. Results and Discussion

Compound **1** was isolated as a white powder. Its molecular formula was established as C_16_H_19_NO_4_ by the positive HR-ESI-MS ions at *m*/*z* 290.13887 [M+H]^+^ (calcd for C_16_H_20_NO_4_, 290.13868), indicating 8 degrees of unsaturation. The ^1^H NMR spectrum (Table 1) displayed two aromatic protons [*δ*_H_ 8.22 (s, H-2); 7.57 (s, H-5)] owing to a 3,4,6-trisubstituted pyridine ring, one olefinic proton [*δ*_H_ 6.40 (s, H-8)], two methines [*δ*_H_ 2.66 (m, H-10); one oxygenated CH *δ*_H_ 3.88 (t, H-2′)], two methylenes [*δ*_H_ 4.25 (t, *J* = 10.8 Hz, H-11a), 4.65 (dd, *J* = 11.1, 5.0 Hz, H-11b); 2.81(dd, *J* = 17.4, 4.5 Hz, H-1′a), 3.08 (dd, *J* = 17.4, 4.5 Hz, H-1′b)], and three methyls [*δ*_H_ 1.18 (d, *J* = 7.0 Hz, H-12); 1.36 (s, H-4′); 1.40 (s, H-5′)]. The ^13^C NMR and DEPT spectral data (Table 1) of **1** displayed the presence of 16 carbons, including eight sp^3^ and eight sp^2^ carbons. Except for five sp^2^ carbons (*δ*_C_ 140.3, 152.4, 127.7, 122.7, 142.3) belonging to the pyridine ring (ring A), the remaining three sp^2^ carbons were classified as one carbonyl group (*δ*_C_ 196.1) and an olefin group (*δ*_C_ 168.2, 101.6). The 1D NMR data and molecular formula indicated that **1** is an amphichoterpenoid type meroterpenoid with a tricyclic ring system [12].

**Figure 3:**
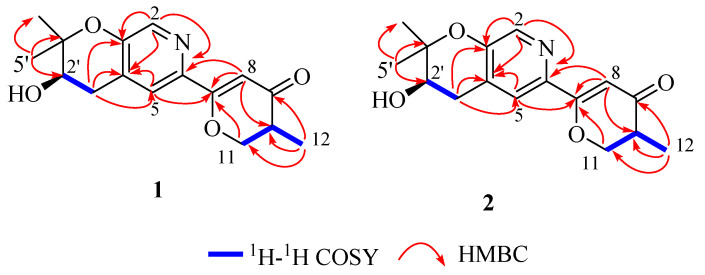
Key ^1^H-^1^H COSY and HMBC correlations of compounds **1** and **2**.

Further analyses of the ^1^H-^1^H COSY and HMBC spectra resulted in the identification of the planar structure of **1**. The ^1^H-^1^H COSY spectrum indicated the presence of two independent spin systems, H_2_-11/H-10/H_3_-12 and H_2_-1′/H-2′ (Figure 3). The HMBC cross-peaks from H-2 to C-3, C-4, and C-6, and from H-5 to C-3, and their chemical shifts, can establish the core fragment of the pyridine ring (unit A). Subsequently, the substructure of the *γ*-pyranone ring (unit B), located at C-6 of unit A, was determined by analyzing the ^1^H-^1^H COSY of H_2_-11/H-10/H_3_-12, and the key HMBC correlations from H-5 to C-7; from H-8 to C-6, C-7, and C-10; from H-11 to C-7; from H_3_-12 to C-9, C-10, and C-11 (Figure 3). The ^1^H-^1^H COSY of H_2_-1′/H-2′ and the HMBC correlations from H_2_-1′ to C-3, C-4, C-5, C-2′, and C-3′; from H_3_-5′ to C-2′, C-3′ and C-4′, H_3_-4′ to C-2′, C-3′ and C-5′ along with the required one degree of unsaturation, revealed a dimethyl-substituted pyran ring (unit C) fused with unit A. Therefore, the resulting planar structure of **1** was established (Figure 3).

**Figure 4:**
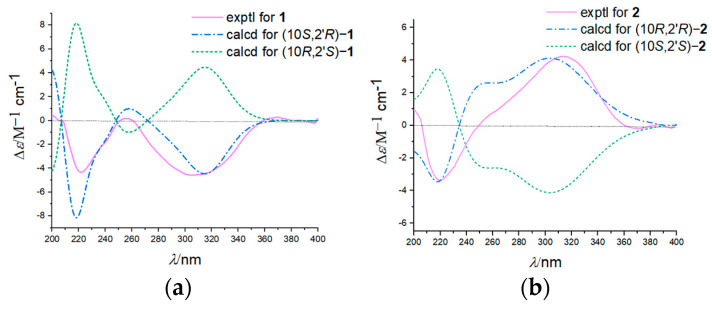
Experimental and calculated ECD spectra of compounds **1** (**a**) and **2** (**b**).

Compound **2** was obtained as a white solid. The molecular formula was assigned as C_16_H_19_NO_4_ based on positive-ion HR-ESI-MS (*m*/*z* 290.13876 [M+H]^+^ (calcd. for C_16_H_20_NO_4_, 290.13868). The detailed analysis of its NMR spectroscopic data (Table 1) revealed that **2** possesses an identical planar structure to **1**, confirmed by the extensive 2D NMR spectroscopic analysis (Figure 3). Based on compounds **1** and **2** purified from the same fraction with different optical rotations, a minor variation (±0.03) of *δ*_C_ (C-10, C-11, and C-12), their NOESY spectrum analysis (Figure S23), and opposite Cotton effects at 320 nm in their experimental ECD spectra (Figure 4), it is speculated that they were 10-epimers.

Compounds **1** and **2** are a pair of epimers exhibiting the same planar structure with only 2 chiral centers at C-10 and C-2′, suggesting four possible configurations (10*S*, 2′*R*), (10*R*, 2′*S*), (10*R*, 2′*R*) and (10*S*, 2′*S*). Thus, the absolute configurations were determined by calculating their theoretical ECD and comparing them to the experimental curves and cotton effect values. The theoretical ECD spectrums were constructed by the time-dependent density functional theory (TDDFT) method at the B3LYP/6–311G** level in methanol. The predicted ECD curves of (10*S*, 2′*R*) −**1** and (10*R*, 2′*R*) −**2** were matched well with the experimental ones (Figure 4). Furthermore, the experimental ECD curve of **1** was close to that of (10*S*, 2′*R*) −amphichoterpenoid B previously reported by Jiang M. et al. using X-ray diffraction, which supported the absolute configurations of **1** as 10*S*, 2′*R* (Figure S24) [12]. So, the absolute configurations of **1** and **2** were determined as 10*S*, 2′*R,* and 10*R*, 2′*R,* respectively. Consequently, the structures of **1** and **2** were established, as shown in Figure 2, and were named amphichoterpenoids D and E. 

Amphichoterpenoids D (**1**) and E (**2**) are the second report of picoline-derived meroterpenoids with 6/6/6 tricyclic pyrano[3,2-c]pyridinyl-*γ*-pyranone scaffold, which may be derived from the lysine-terpenoid-polyketone hybrid biosynthetic pathway [12]. This pair of epimers (**1**, **2**) are the direct biogenic precursors of amphichoterpenoids A−C. This study added members of the rare class of picoline-derived meroterpenoids [12].

**Figure 5:**
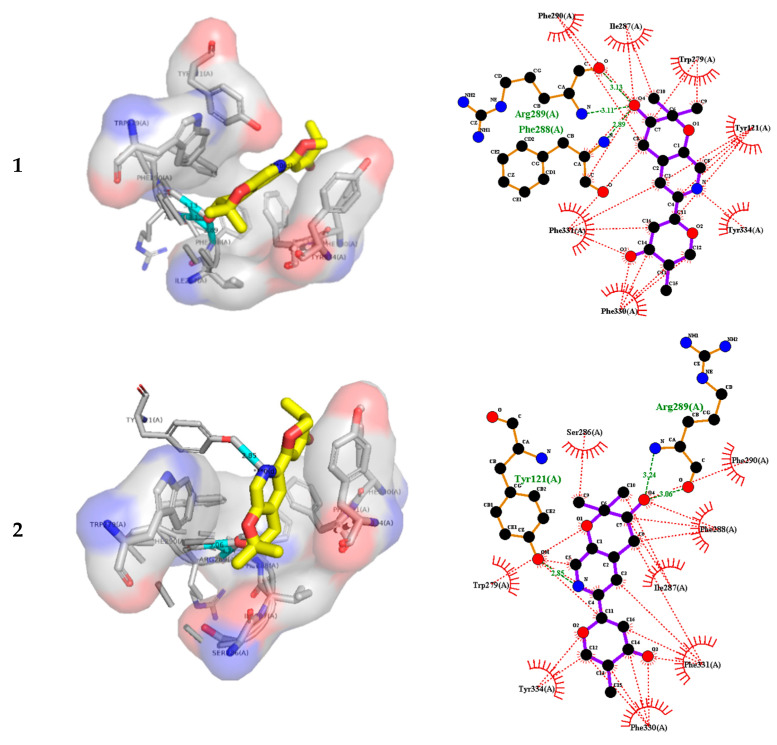

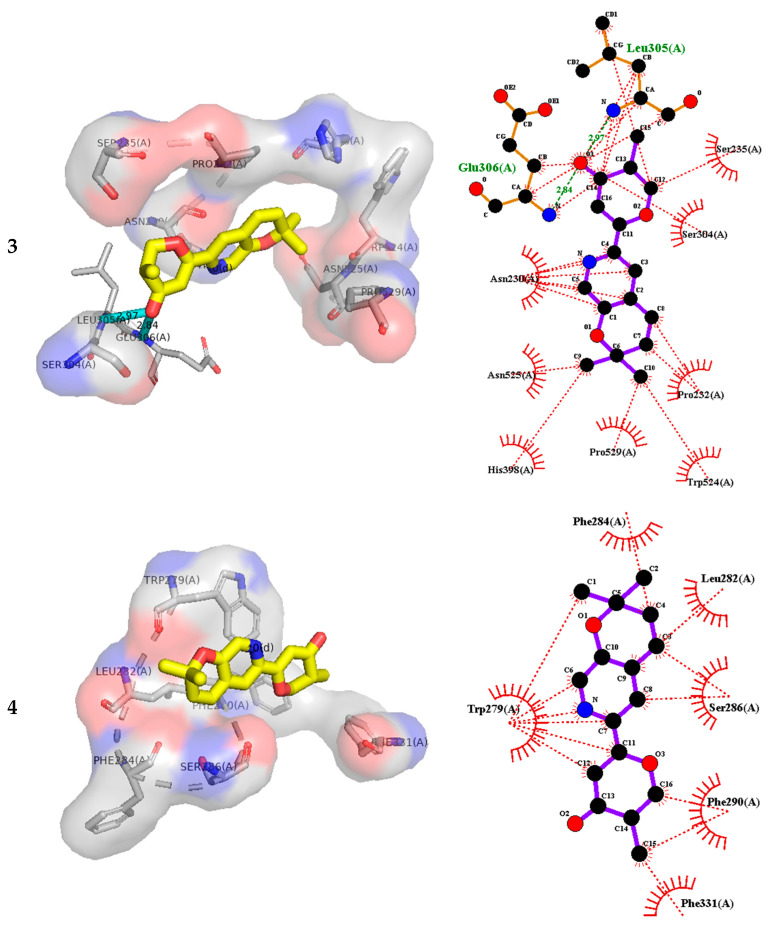
Docking model for compounds **1**−**4** with AChE (PDB ID: 1QTI). Hydrogen bonds and hydrophobic interactions are indicated by green and red lines between the atoms involved, respectively.

The acetylcholinesterase (AChE) inhibitor is the primary drug target for treating Alzheimer’s disease [10,32,33]. The huperzine A, physostigmine, berberine, and marketed drugs (galanthamine and rivastigmine) were representative natural products derived-AchE reversible inhibitors with significant activity [34,35,36]. Here, the AChE inhibitory activities of compounds **1** and **2** were evaluated by Ellman’s method and using rivastigmine as the positive control [12,37]. Compounds **1** and **2** exhibited AChE inhibitory activity with IC_50_ values of 12.5 μM and 11.6 μM, respectively, significantly less active than the positive control, rivastigmine (IC_50_, 3.9 μM). About 38% of the naturally-derived alkaloids (55 molecules) were considered potential AChE inhibitors with an IC_50_ ≤ 10 μM [34,35]. Here, we have added a new class of natural meroterpenoid alkaloids for AChE inhibitors. Besides, because compounds **1**, **2**, and (+)/(−)-amphichoterpenoids A (**3**, **4**) (Figure 2) [12] have the same planar structure but quite different anti-AChE activities in vitro (Table S3), molecular docking analysis was performed to investigate the mechanism of the inhibitory effects of amphichoterpenoids on the AChE enzyme (PDB ID: 1QTI). The results (Figure 5, Table 2) suggested that compounds **1**–**4** matched well in the protein-binding pocket of AChE protein, but different interactions with AChE were found. Generally, the low binding energy indicats that the active compound is easily bound to the protein. The binding energy of the AChE enzyme and **1** was −9.3 kcal mol^−^^1^, with three hydrogen bonds and two interacting residues, Arg289 and Phe288, which was similar to that of **2** (Figure 4, Table 2). However, the binding affinity between **3** and the AChE enzyme was −7.9 kcal mol^−^^1^, with two hydrogen bonds and two interaction residues, Leu305 and Glu306, that was higher than that of **1, 2,** and lower than that of **4** (binding energy: −6.8 kcal mol^−^^1^, without hydrogen bond) (Figure 5, Table 2). It is well known that there are four subsites in the inhibitor-binding gorge-like pocket of AChE, including the catalytic active site (CAS, including Ser200, His440, and G1u327), the peripheral anionic site (PAS, including Tyr70, Tyr121, Trp279, and Asp72), the hydrophobic site (or choline-binding site, including Trp84, Glu199, Phe330, and Tyr442), and the acyl pocket (including Phe288 and Phe290). The binding site between the compounds **1**, **2** and AChE is not CAS but other active sites (Tyr121 and Trp279 residues in the PAS, Phe330 residue in the hydrophobic site, and Phe288 and Phe290 residues in the acyl pocket) that play an important role in the enzyme activity, while compound **3** can interact with some amino acid residues at the substrate binding site (not CAS or PAS) in the pocket of AChE to form hydrogen bonds and hydrophobic interactions, which partially inhibit AChE activity. These results further supported the different acetylcholine inhibitory activities of **1**−**4** in vitro.

## 3. Materials and Methods

### 3.1. General Experimental Procedures

Optical rotations were carried out on an MCP 200 (Anton Paar, Graz, Austria) polarimeter. UV spectra were measured at a Lambda 950 UV-Vis-NIR spectrophotometer (PerkinElmer, Akron, OH, USA). A Chirascan-plus Circular Dichroism Spectrometer (Applied Photophysics Ltd., Leatherhead, UK) was used to obtain experimental ECD data. A Fourier transformation infra-red spectrometer (FTIR) coupled with an infra-red microscope EQUINOX 55 (Bruker, Wissembourg, France) recorded the FTIR spectrum. NMR spectra were tested by a BRUKER AVANCE III HD (400 MHz) NMR spectrometer with tetramethylsilane (TMS) as the internal standard. HR-ESIMS data were determined using an Agilent 6530 accurate-Mass Q-TOF LC-MS spectrometer. Column chromatography (CC) was used using silica gel (200–300 mesh, Qingdao Marine Chemical Factory, Qingdao, China). The semi-preparative HPLC was performed on an Essentia LC-16 (Shimadzu, Jiangsu, China). Acetylcholine esterase (AChE) was from Electrophorus electricus (product number: C3389-2KU, Sigma-Aldrich, Saint Louis, MO, USA).

### 3.2. Fungal Material

The strain was identified as *Amphichorda felina* (syn. *Beauveria felina*) SYSU-MS7908 based on the rDNA ITS sequence (GenBank NO. MT786206) [12]. The strain was preserved at Guangdong Microbial Culture Collection Center (GDMCC NO. 61059) and the School of Marine Sciences, Sun Yat-Sen University.

### 3.3. Extraction and Isolation

The strain *A. felina* SYSU-MS7908 was grown on Yeast extract Peptone Dextrose Agar at 26 °C. Then it was cut into pieces and cultivated on rice medium (40 mL rice, 40 mL water with 3% artificial sea salt, and 0.3% peptone) in 200 flasks for 28 days at room temperature. The solid fermented substrate was extracted exhaustively with MeOH three times to obtain a crude extract, then suspended in water and continuously extracted three times with EtOAc. The EtOAc extract (170 g) was fractionated to CC on silica gel (200–300 mesh) and was eluted with petroleum ether/EtOAc of increasing polarity (from 9:1 to 0:10) to obtain six fractions (A–F).

Fr.C was fractionated on a Sephadex LH-20 column with MeOH/CH_2_Cl_2_ (1:1) to afford three fractions (Fr.C.1 to Fr.C.3). Fr.C.2 was further fractionated by RP-HPLC (MeOH /H_2_O, 65:35 flow rate 2 mL/min, ACE-C18-AR column 10 × 250 mm, 5 μm) to give subfraction (Fr.C.2.4). Fr.C.2.4 was further purified by RP-HPLC (MeOH/H_2_O, 55:45 flow rate 2 mL/min, ACE-C18-PFP column 10 × 250 mm, 5 μm) to give **1** (3.0 mg) and **2** (2.7 mg). 

Amphichoterpenoid D (**1**)**:** White powder; mp 125–128 °C; ${\left[\alpha \right]}_{D}^{20}$ −69.8 (*c* 0.20, MeOH); CD (MeOH) *λ*_max_ (Δ*ε*) 220 (−4.10), 318 (−4.15) nm; UV (MeOH) *λ*_max_ (log *ε*) 220 (1.95), 319 (3.24) nm; IR (neat) *v*_max_ 3359, 2980, 2918, 1643, 1606, 1556, 1376, 1125, 1058, cm^−1^; ^1^H NMR (CDCl_3_, 400 MHz) and ^13^C NMR (CDCl_3_, 100 MHz) data, see Table 1; HR-ESIMS *m*/*z* 290.13887 [M+H]^+^ (calcd for C_16_H_20_NO_4_, 290.13868).

Amphichoterpenoid E (**2**)**:** White powder; mp 131−134 °C; ${\left[\alpha \right]}_{D}^{20}$ +57.6 (*c* 0.20, MeOH); CD (MeOH) *λ*_max_ (Δ*ε*) 220 (−3.92), 320 (+4.21) nm; UV (MeOH) *λ*_max_ (log *ε*) 220 (1.82), 319 (3.25) nm; IR (neat) *v*_max_ 3359, 2980, 2918, 1643, 1606, 1556, 1376, 1125, 1058, cm^−1^; ^1^H NMR (CDCl_3_, 400 MHz) and ^13^C NMR (CDCl_3_, 100 MHz) data, see Table 1; HR-ESIMS *m*/*z* 290.13876 [M+H]^+^ (calcd for C_16_H_20_NO_4_, 290.13868).

### 3.4. Calculation of the ECD Spectra

Molecular Merck force field (MMFF) and TDDFT ECD calculations were performed with Spartan’14 software package (Wavefunction Inc., Irvine, CA, USA) and Gaussian 09 program package, respectively, using default grids and convergence criteria. MMFF conformational search generated low-energy conformers within a 10 kcal/mol energy window were subjected to geometry optimization using the DFT method at the B3LYP/6–31 G(d, p) in gas. Frequency calculations were run at the same condition to estimate their relative thermal free energies (Δ*G*) at 298.15 K. Energies of the low-energy conformers with Boltzmann distribution over 1% in MeOH were re-calculated at the B3LYP/6–311G** level. Solvent effects were taken into account by using an IEF-PCM model. The TDDFT calculations were performed in MeOH using the B3LYP/6–311G** level for all conformers. Rotatory strengths for a total of 20–50 excited states were calculated. The ECD spectra were produced by the programs SpecDis 1.6 (University of Würzburg, Würzburg, Germany) and OriginPro 8.5 (OriginLab, Ltd., Northampton, MA, USA) using a Gaussian band shape from dipole-length dipolar and rotational strengths with 0.30 eV exponential half-width. The equilibrium population of every conformer at 298.15 K was calculated from its relative free energies using Boltzmann statistics. The calculated spectra of **1** and **2** were generated from the low-energy conformers according to the Boltzmann distribution of each conformer in the MeOH solution. All calculations were performed by Tianhe-2 in National Super Computer Center in Guangzhou.

### 3.5. Anti-Acetylcholinesterase Activity

The acetylcholinesterase (AChE) inhibitory activity of compounds **1** and **2** was evaluated by the modified Ellman’s method with rivastigmine as a positive control [12,37]. All experiments were performed in triplicate. The detailed experiment was shown in supporting information.

### 3.6. Molecular Docking

The binding interaction between compounds **1**–**4** and AChE enzyme at the active site was investigated by molecular docking. Their 3D structures were optimized to establish the lowest energy state and saved in mol.2 file format by chem3D 16.0 software. The protein crystallographic structure of the AChE (PDB ID: 1QTI) was selected from the RCSB Protein Data Bank with 2.5 Å resolution [38]. Molecular docking was conducted using Autodocktools-1.5.6 and PyMOL-2.3.4. Autodock Vina-1.2 [39] was used to study the interaction. PyMOL-2.3.4 and LigPlot + were applied to analyze the result of the binding mode.

## 4. Conclusions

The chemical investigation of the ascidian-derived fungus *A. felina* SYSU-MS7908 afforded a pair of new picoline-derived meroterpenoid epimers, amphichoterpenoids D (**1**) and E (**2**), which possess a 6/6/6 tricyclic pyrano[3,2-c]pyridinyl-*γ*-pyranone scaffold. This study enriched the members of the following rare class of picoline-derived meroterpenoid: amphichoterpenoids. Moreover, **1** and **2** showed potential AChE inhibitory activity, indicating its potential use in the treatment of Alzheimer’s disease.

## Figures and Tables

**Table 1:** ^1^H (400 MHz) and ^13^C (100 MHz) NMR data for compounds **1** and **2** (CDCl_3_).

No.	1		2	
	*δ*_C_, Type	*δ*_H_, Mult (*J* in Hz)	*δ*_C_, Type	*δ*_H_, Mult (*J* in Hz)
2	140.3, CH	8.22, s	140.3, CH	8.22, s
3	152.4, C		152.4, C	
4	127.7, C		127.6, C	
5	122.7, CH	7.57, s	122.7, CH	7.57, s
6	142.3, C		142.3, C	
7	168.2, C		167.9, C	
8	101.6, CH	6.40, s	101.6, CH	6.40, s
9	196.1, C		196.1, C	
10	39.27, CH	2.66, m	39.27, CH	2.67, m
11	73.68, CH_2_	a:4.65, dd (11.1, 5.0); b: 4.25, t (10.8)	73.69, CH_2_	a:4.64, dd (11.1, 5.0); b: 4.25, t (10.8)
12	11.34, CH_3_	1.18, d (7.0)	11.37, CH_3_	1.18, d (7.0)
1′	30.9, CH_2_	a: 3.08 dd (17.4, 4.5); b: 2.81 dd (17.4, 4.5)	30.9, CH_2_	a: 3.08 dd (17.4, 4.5); b: 2.81 dd (17.4, 4.5)
2′	68.9, CH	3.88, t (9.9)	68.9, CH	3.89, t (9.9)
3′	78.7, C		78.6, C	
4′	25.0, CH_3_	1.36, s	25.0, CH_3_	1.36, s
5′	22.1, CH_3_	1.40, s	22.1, CH_3_	1.40, s

**Table 2:** Binding energies and targeting residues in the active pocket between compounds **1**−**4** and AChE (PDB ID: 1QTI).

Compound	log (FBE), kcal/mol	Targeting Residues (H bond Å)	Hydrophobic Interaction Residues
**1**	−9.3	Arg289(3.13,3.11),Phe288(2.89)	Trp279,Tyr121, Phe330, Phe288,Phe290,Ile287, Tyr334, Phe331
**2**	−9.3	Arg289(3.24,3.06),Tyr121(2.85)	Trp279,Tyr121, Phe330, Phe288,Phe290,Ile287, Tyr334, Phe331,Ser286
**3**	−7.9	Leu305(2.97), Glu306(2.84)	Leu305,Glu306,Ser235,Ser304,Pro232,Trp524,Pro529, His398,Asn525,Asn230
**4**	−6.8	none	Trp279, Phe290,Phe284,Leu282,Ser286, Phe331

## Data Availability

Not applicable.

## Supplementary Materials

The following supporting information can be downloaded at: https://www.mdpi.com/article/10.3390/molecules27165076/s1, Figure S1: The HRESIMS spectrum of compound **1**, Figures S2–S8: The 1D and 2D NMR (400 MHz) spectra of compound **1** in CDCl_3_, Figures S9 and S10: The IR/UV spectrum of compound **1**, Figure S11: The HRESIMS spectrum of compound **2**, Figures S12–S18: The 1D and 2D NMR (400 MHz) spectra of compound **2** in CDCl_3_, Figures S19 and S20: The IR/UV spectrum of compound **2**. Figures S21 and S22 and Tables S1 and S2: The energy analysis and low-energy conformers of compounds **1** and **2**. Figure S23: Key NOE correlations of compounds 1 and 2; Figure S24: The X-ray Single crystal structure of amphichoterpenoid B (**5**) and its ECD spectra. Table S3: Inhibitory activity of compounds **1**–**5** on AChE.

## Sample Availability

Samples of the compounds **1** and **2** are available from the authors.
