# Supplementary material for: Two New Picoline-Derived Meroterpenoids with Anti-Acetylcholinesterase Activity from Ascidian-Derived Fungus Amphichorda felina
Source: Molecules. 2022 Aug 10;27(16):5076. doi: 10.3390/molecules27165076 (PMC9416303; doi:10.3390/molecules27165076)
Supplement: Supplementary file 1 [file molecules-27-05076-s001.zip › molecules-1794147-supplementary.pdf]

Supplementary Material

Two New Picoline-Derived Meroterpenoids with Anti-Acetylcholinesterase Activity from Ascidian-Derived Fungus *Amphichorda felina*

Minghua Jiang [1,2], Heng Guo [1,2], Qilin Wu [1,2], Siwen Yuan [1,2] and Lan Liu [1,2,3,*]

[1] School of Marine Sciences, Sun Yat-sen University, Zhuhai 519082, China; jiangmh23@mail2.sysu.edu.cn (M.J.); guoh59@mail2.sysu.edu.cn (H.G.); wuqlin3@mail2.sysu.edu.cn (Q.W.); yuansw@mail2.sysu.edu.cn (S.Y.)

[2] Southern Marine Science and Engineering Guangdong Laboratory (Zhuhai), Zhuhai 519000, China

[3] Pearl River Estuary Marine Ecosystem Research Station, Ministry of Education, Zhuhai 519082, China

* Correspondence: cesllan@mail.sysu.edu.cn

Table of Contents

Figure S1. The HR-ESIMS spectrum of compound **1**. 2

Figure S2. The ^{1}H NMR (400MHz) spectrum of compound **1** in $CDCl_3$ 3

Figure S3. The ^{13}C NMR (100MHz) spectrum of compound **1** in $CDCl_3$. 3

Figure S4. The DEPT135 spectrum of compound **1** in $CDCl_3$. 4

Figure S5. The HSQC spectrum of compound **1** in $CDCl_3$. 4

Figure S6. The ^{1}H-^{1}H COSY spectrum of compound **1** in $CDCl_3$. 5

Figure S7. The HMBC spectrum of compound **1** in $CDCl_3$. 5

Figure S8. The NOESY spectrum of compound **1** in $CDCl_3$ 6

Figure S9. The IR spectrum of compound **1** 6

Figure S10. The UV spectrum of compound **1** 7

Figure S11. The HR-ESIMS spectrum of compound **2**. 7

Figure S12. The ^{1}H NMR (400MHz) spectrum of compound **2** in $CDCl_3$. 8

Figure S13. The ^{13}C NMR (100MHz) spectrum of compound **2** in $CDCl_3$. 8

Figure S14. The DEPT135 spectrum of compound **2** in $CDCl_3$. 9

Figure S15. The HSQC spectrum of compound **2** in $CDCl_3$. 9

Figure S16. The ^{1}H-^{1}H COSY spectrum of compound **2** in $CDCl_3$. 10

Figure S17. The HMBC spectrum of compound **2** in $CDCl_3$. 10

Figure S18. The NOESY spectrum of compound **2** in $CDCl_3$. 11

Figure S19. The IR spectrum of compound **2** 11

Figure S20. The UV spectrum of compound **2** 12

Table S1. Energy Analysis for the Conformers of (10*S*, 2′*R*)-1. 12
Figure S21. B3LYP/6-311g** optimized low-energy conformers of (10*S*, 2′*R*)-1. 12
Table S2. Energy Analysis for the Conformers of (10*R*, 2′*R*)-2. 13
Figure S22. B3LYP/6-311g** optimized low-energy conformers of (10*R*, 2′*R*)-2. 13
Figure S23. Key NOE (purple dash arrow) correlations of compounds 1 and 2 14
Figure S24. The X-ray Single crystal structure of amphichoterpenoid B (5) and its ECD spectra [1]. 14
Table S3. Inhibitory activity of compounds 1-5 on AChE 14

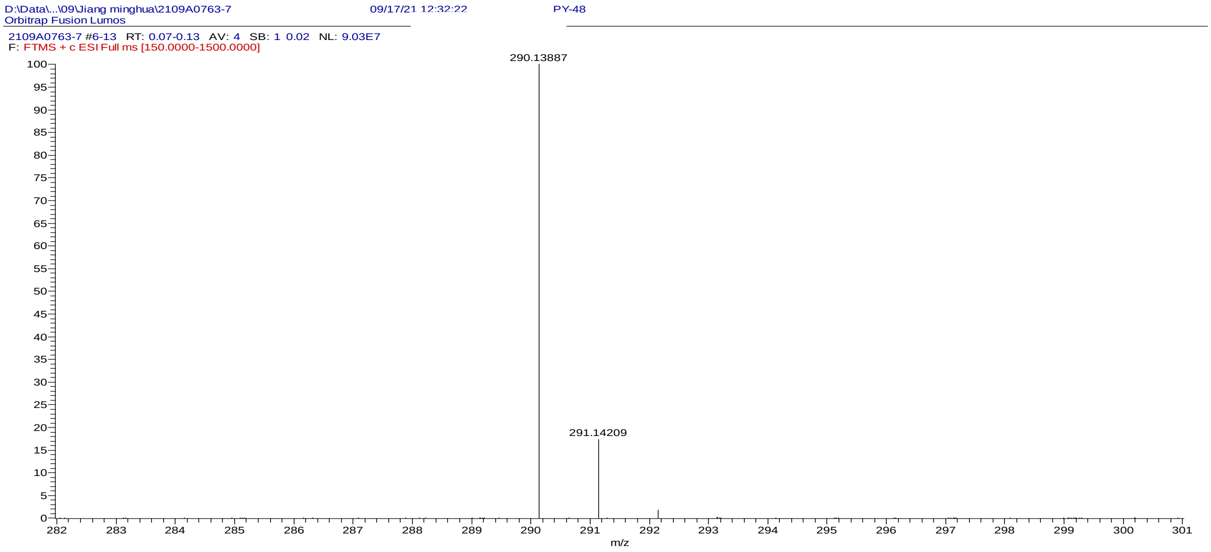

SPECTRUM -
simulation :

m/z	Theo. Mass	Delta (ppm)	RDB equiv.	Composition
290.13887	290.13868	0.64	7.5	$C_{16}H_{20}O_4N$

Figure S1. The HR-ESIMS spectrum of compound **1**.

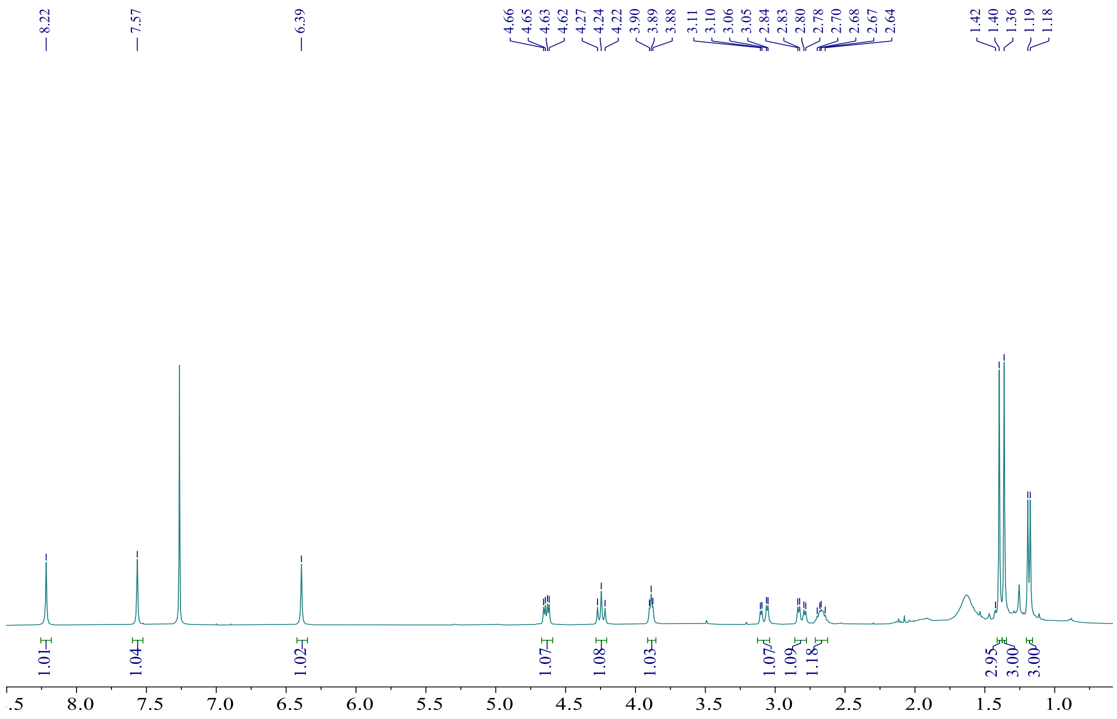

Figure S2. The ^{1}H NMR (400MHz) spectrum of compound **1** in $CDCl_3$

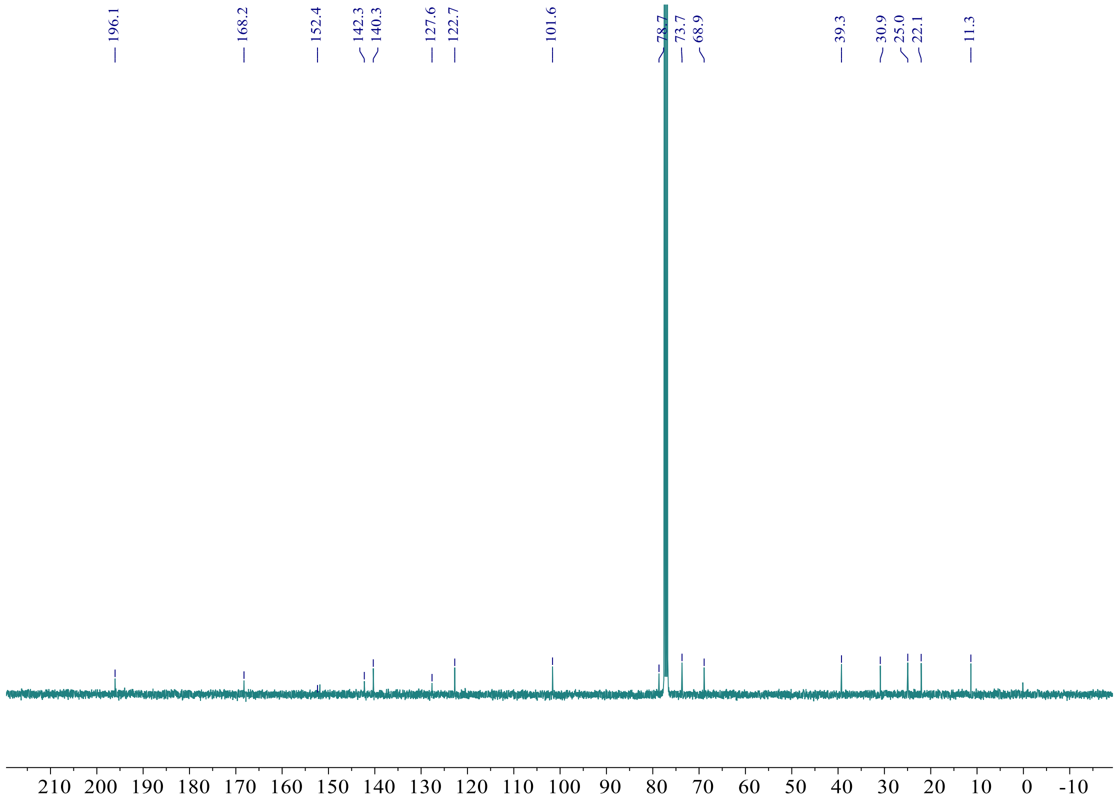

Figure S3. The ^{13}C NMR (100MHz) spectrum of compound **1** in $CDCl_3$.

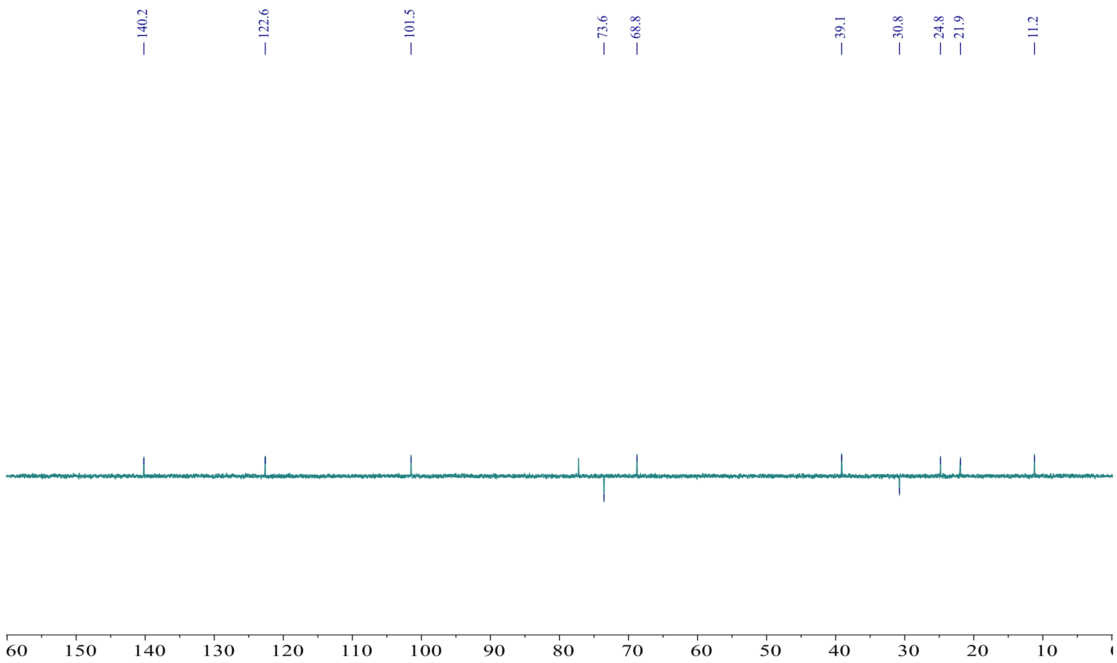

Figure S4. The DEPT135 spectrum of compound **1** in $CDCl_3$.

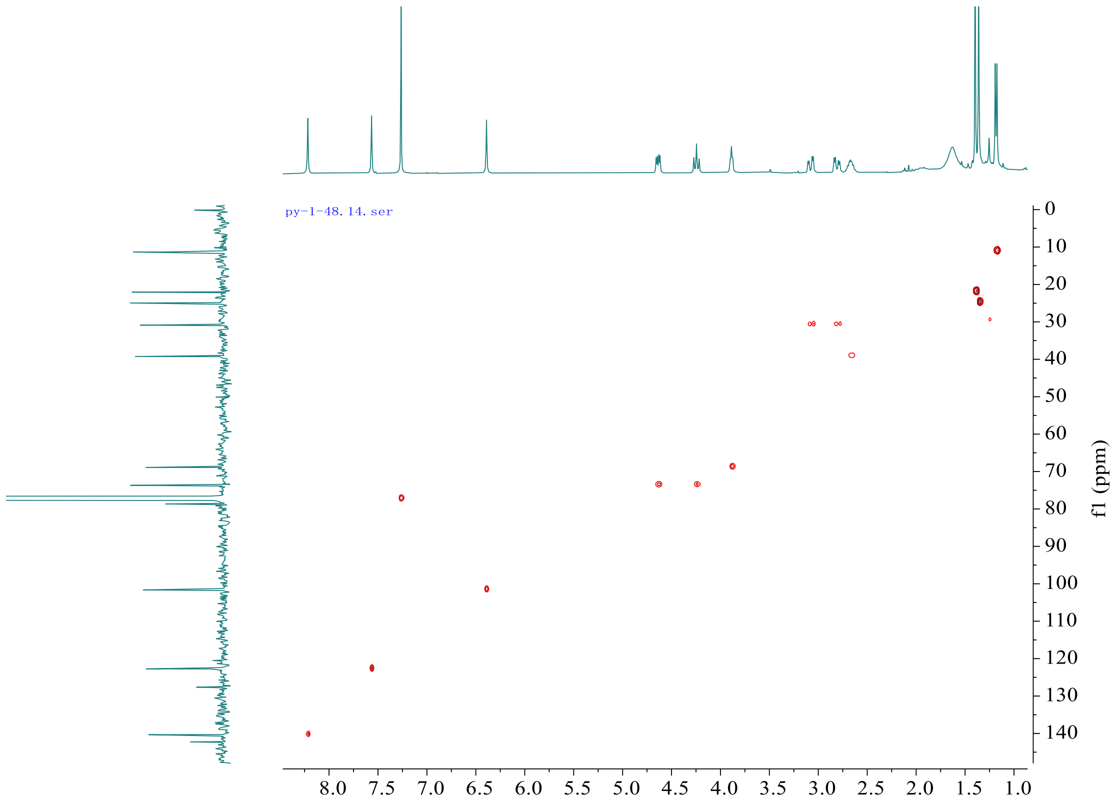

Figure S5. The HSQC spectrum of compound **1** in $CDCl_3$.

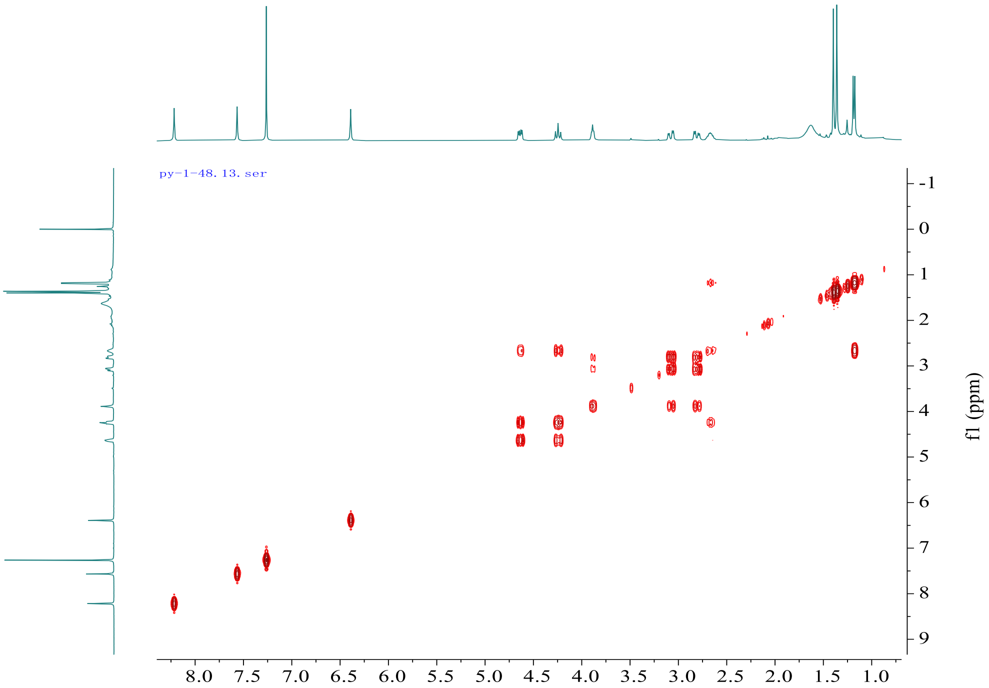

Figure S6. The ^1H-^1H COSY spectrum of compound **1** in $CDCl_3$.

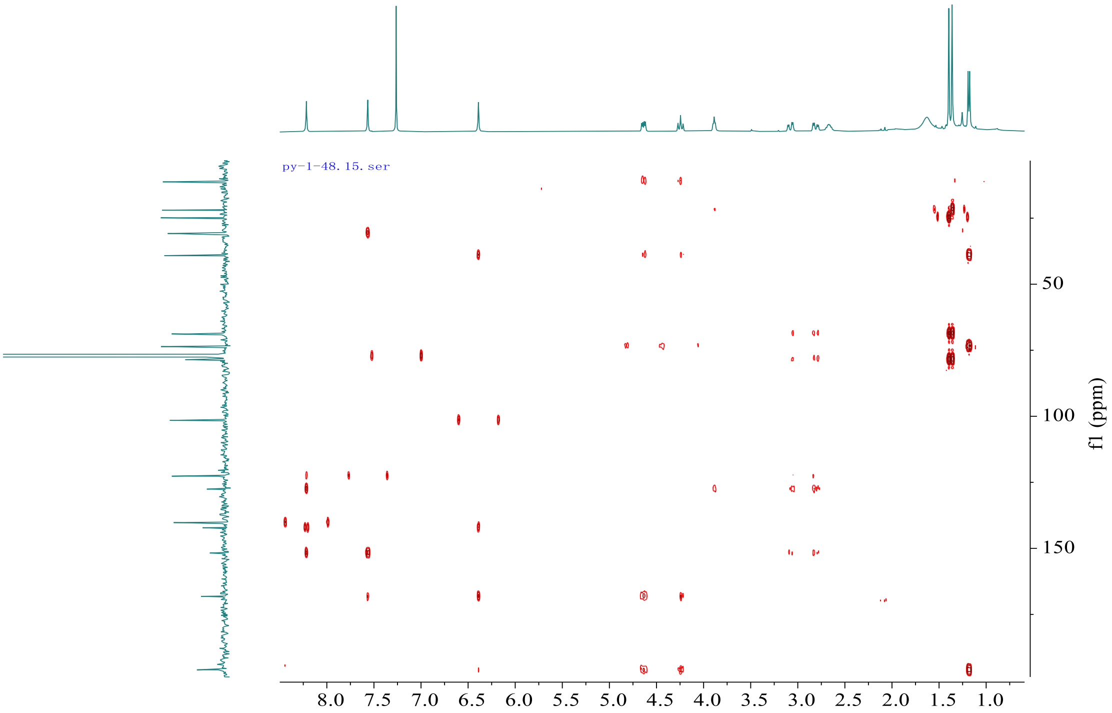

Figure S7. The HMBC spectrum of compound **1** in $CDCl_3$.

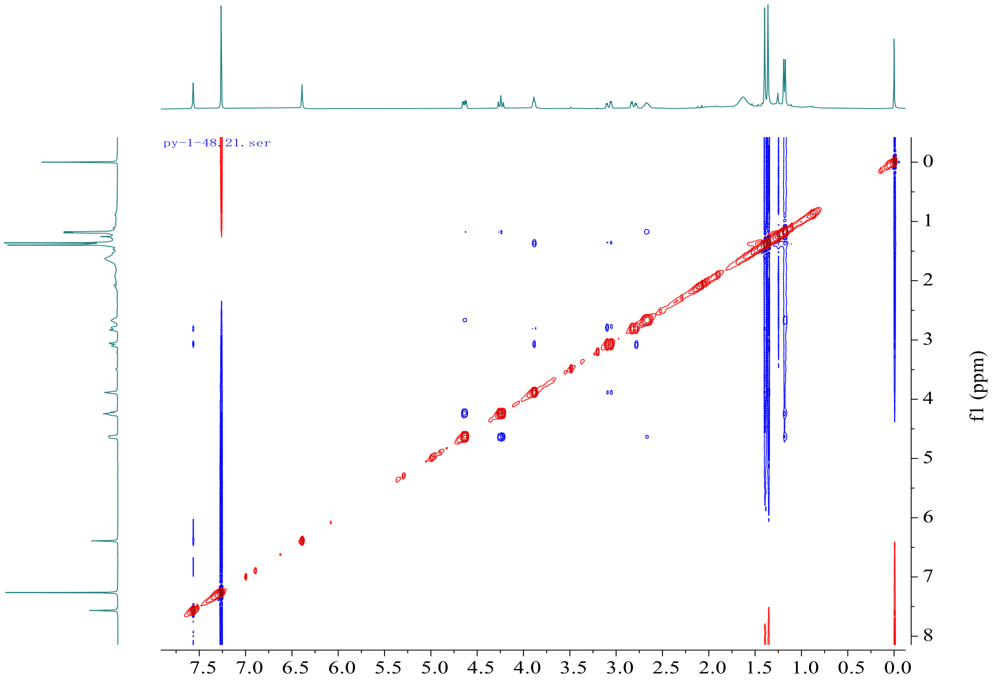

Figure S8. The NOESY spectrum of compound **1** in $CDCl_3$

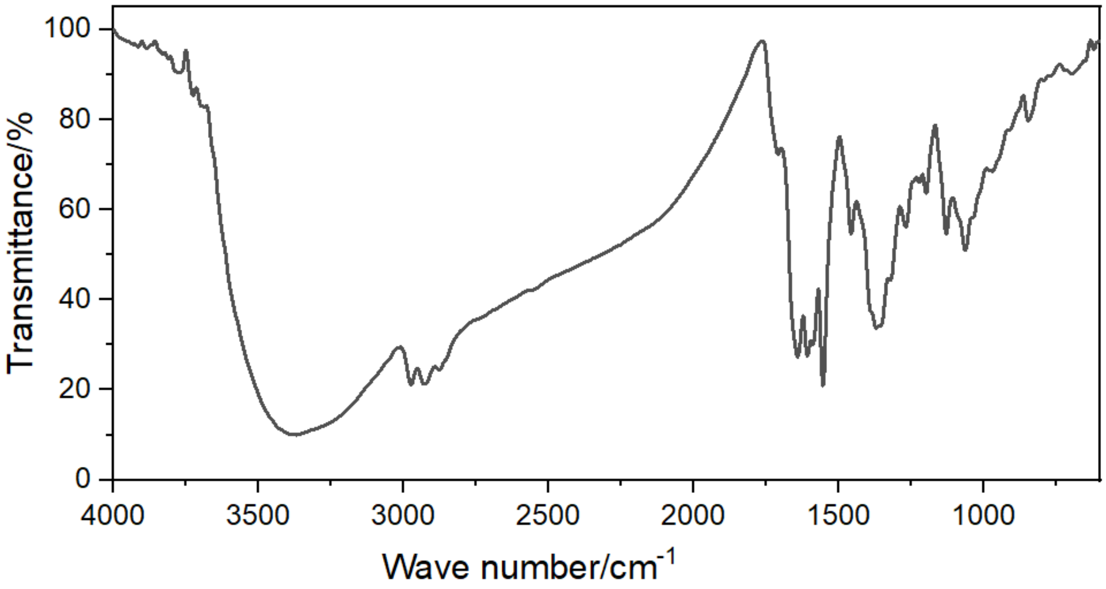

Figure S9. The IR spectrum of compound **1**

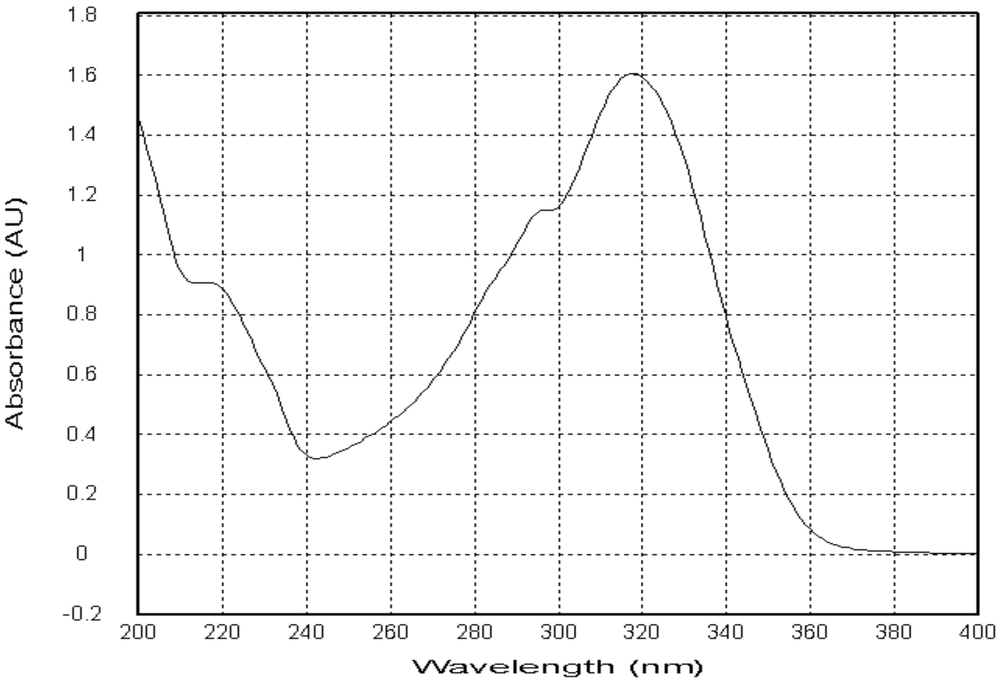

Figure S10. The UV spectrum of compound **1**

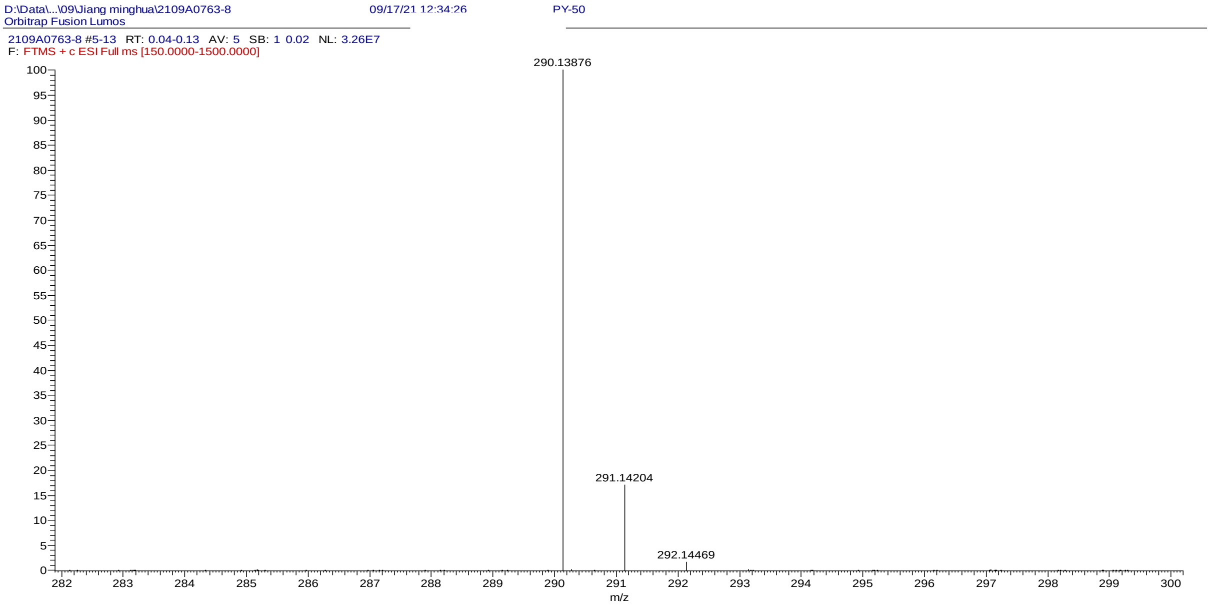

SPECTRUM - simulation :

m/z	Theo. Mass	Delta (ppm)	RDB equiv.	Composition
290.13876	290.13868	0.26	7.5	$C_{16} H_{20} O_4 N$

Figure S11. The HR-ESIMS spectrum of compound **2**.

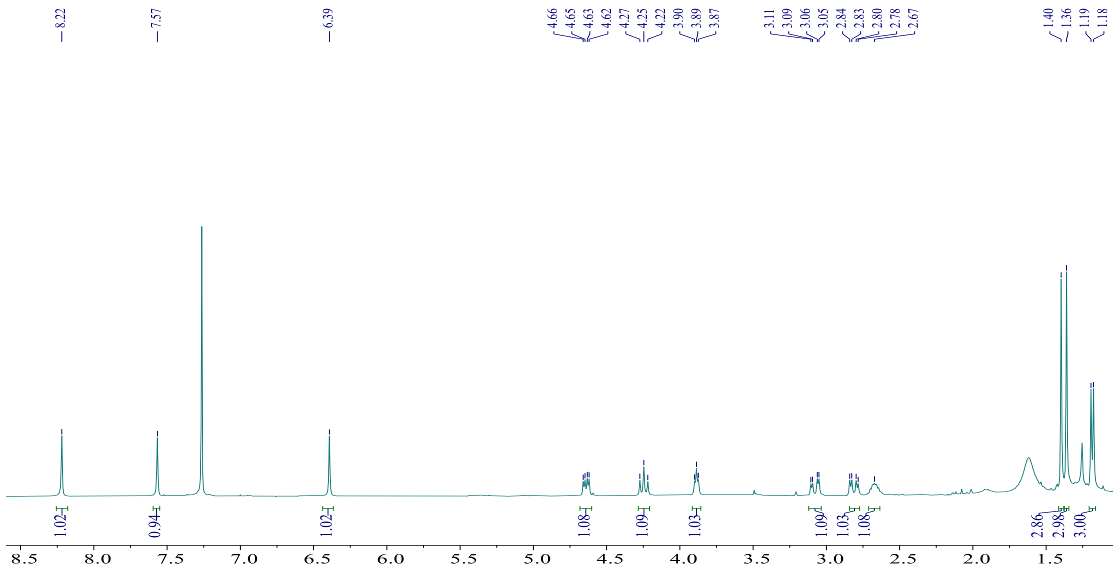

Figure S12. The ^{1}H NMR (400MHz) spectrum of compound **2** in $CDCl_3$.

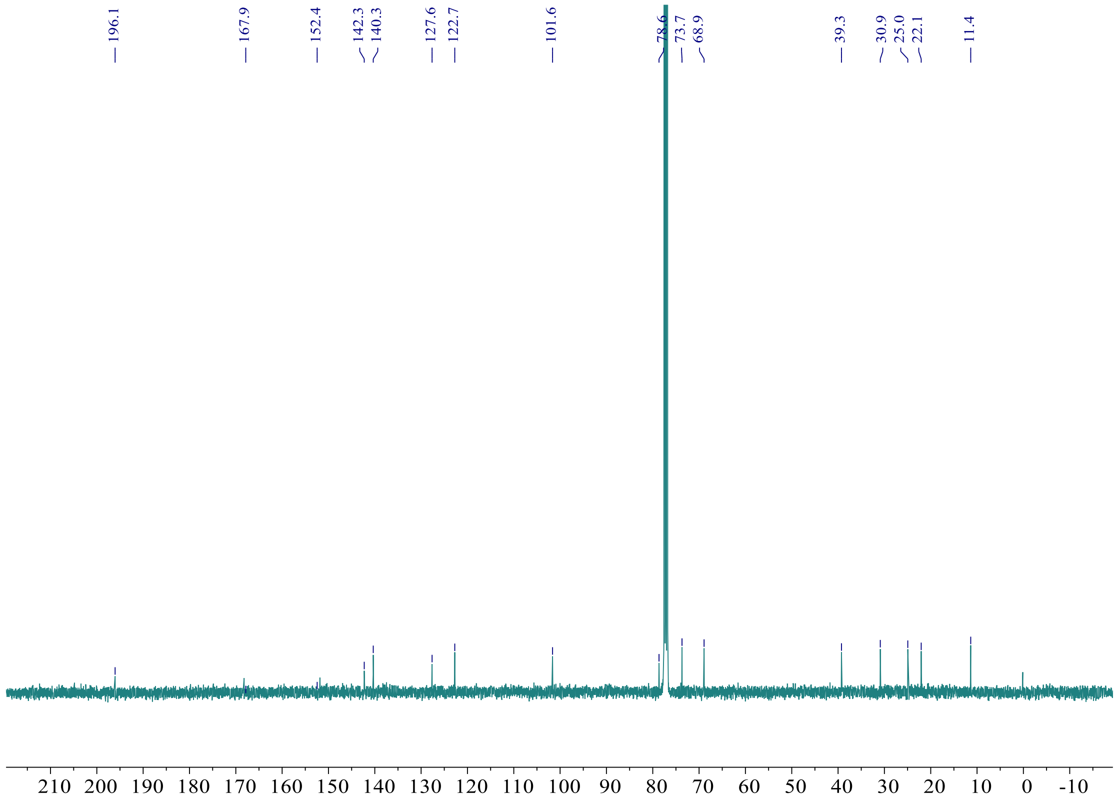

Figure S13. The ^{13}C NMR (100MHz) spectrum of compound **2** in $CDCl_3$.

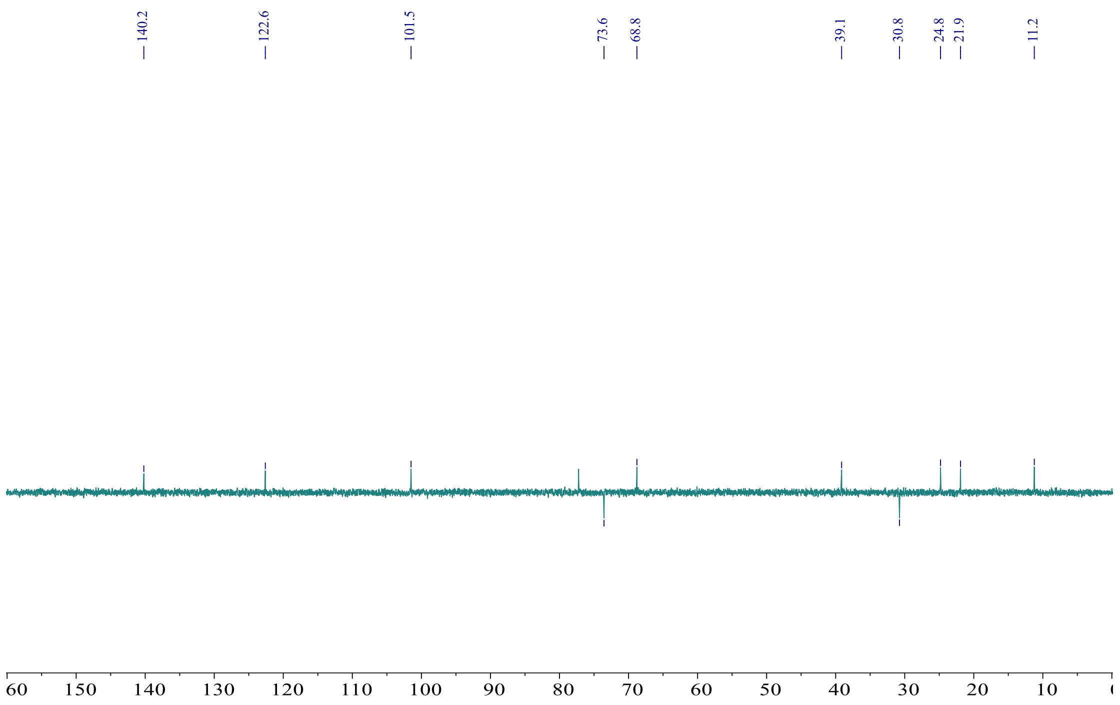

Figure S14. The DEPT135 spectrum of compound **2** in $CDCl_3$.

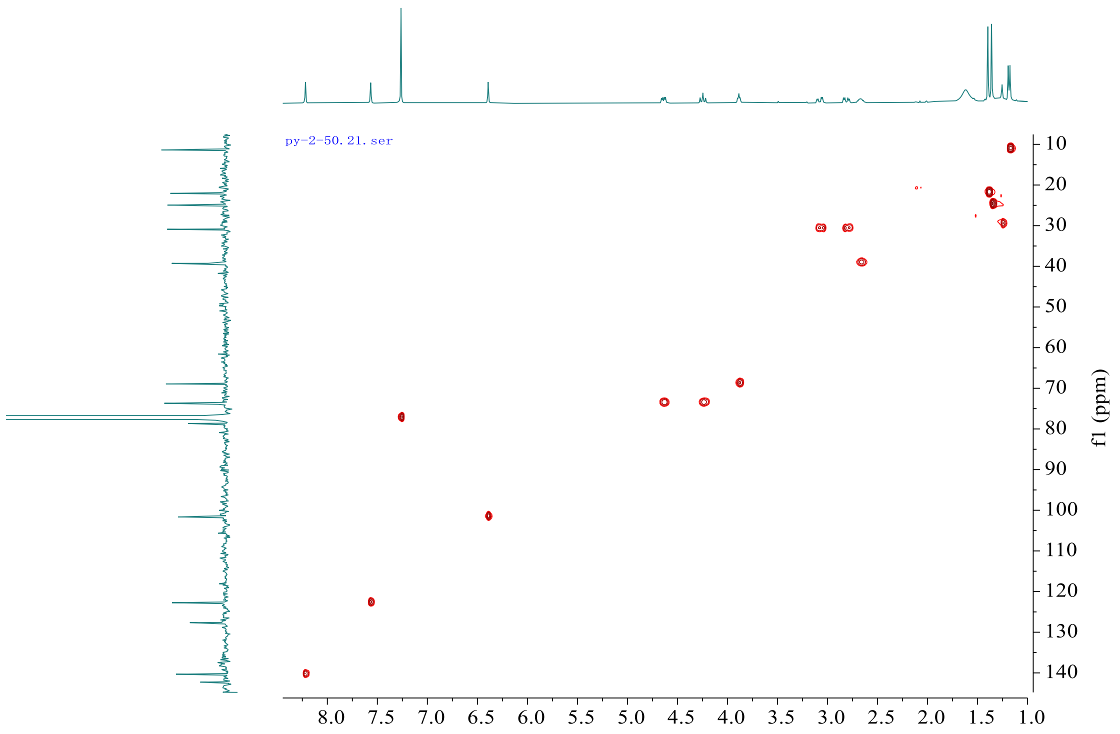

Figure S15. The HSQC spectrum of compound **2** in $CDCl_3$.

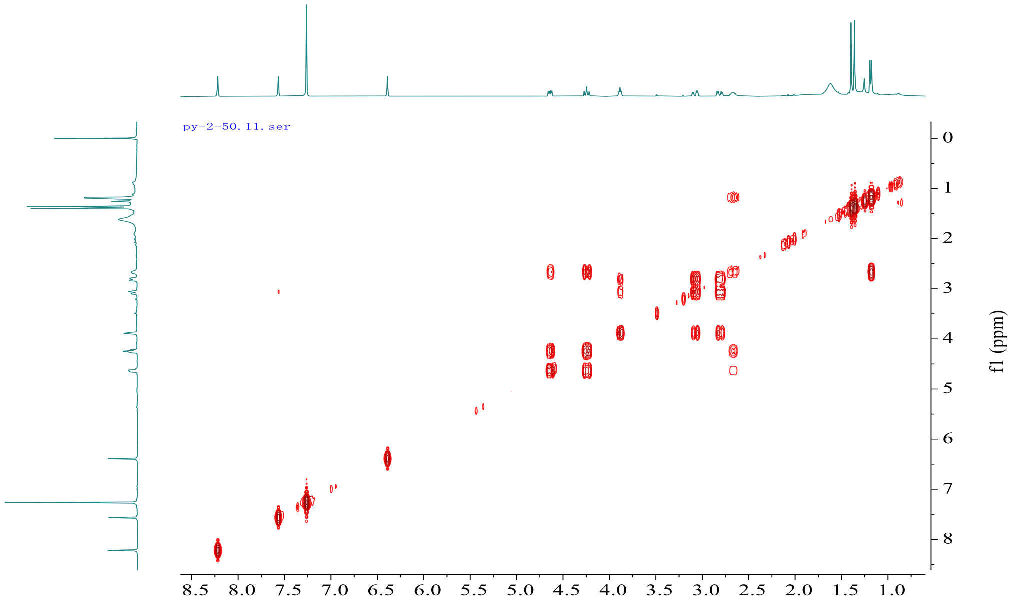

Figure S16. The ^{1}H-^{1}H COSY spectrum of compound **2** in $CDCl_3$.

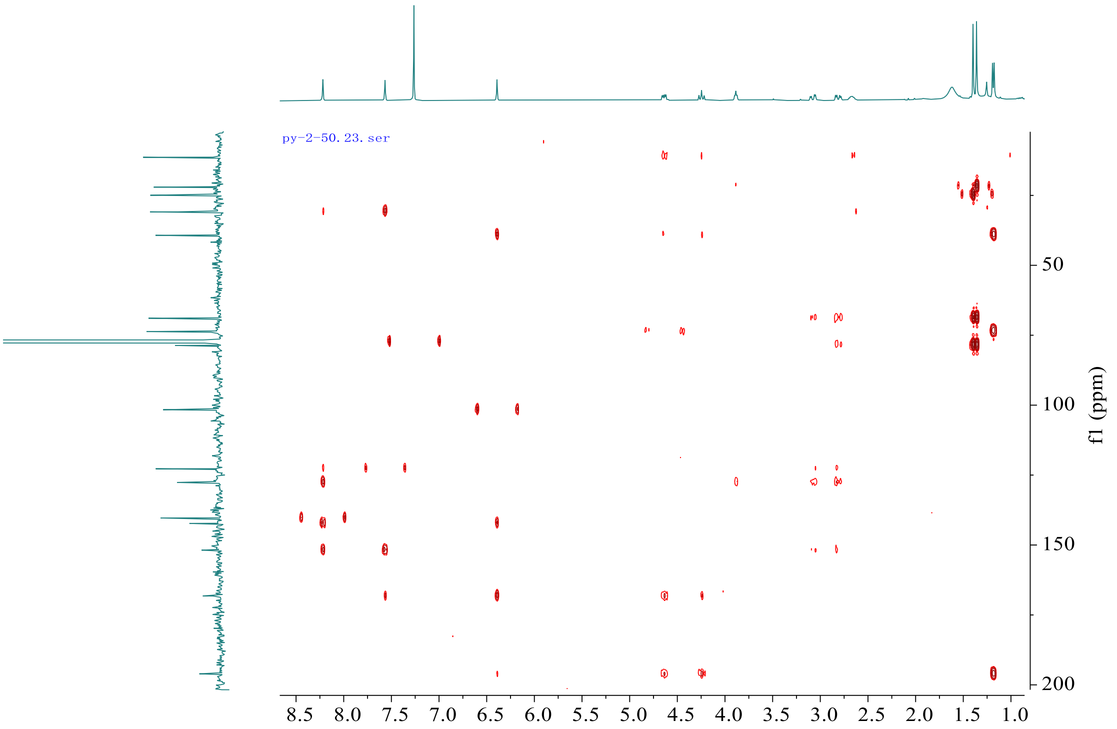

Figure S17. The HMBC spectrum of compound **2** in $CDCl_3$.

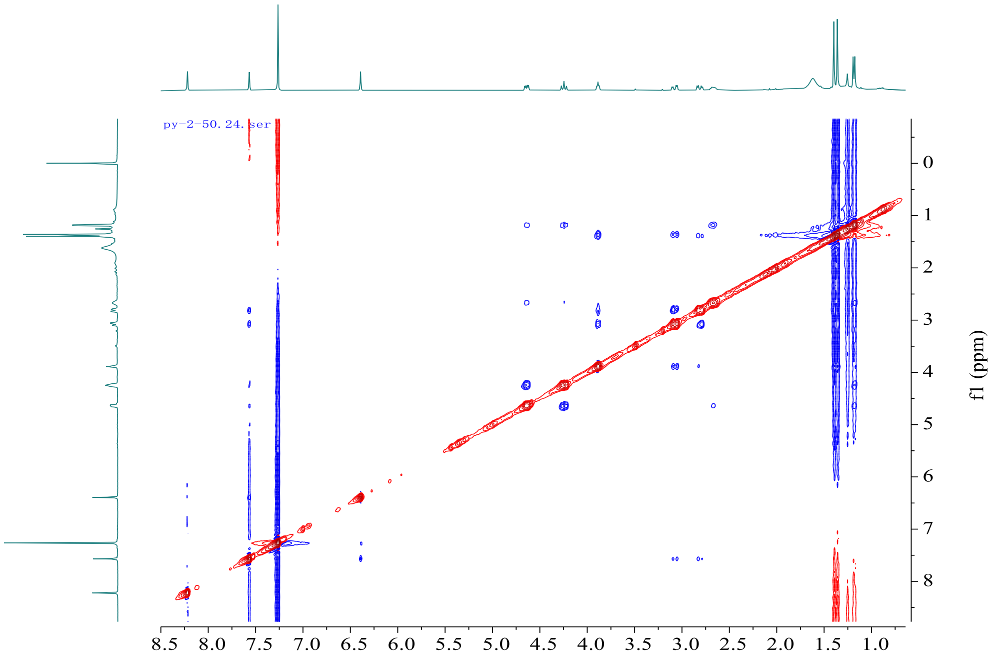

Figure S18. The NOESY spectrum of compound **2** in CDCl3.

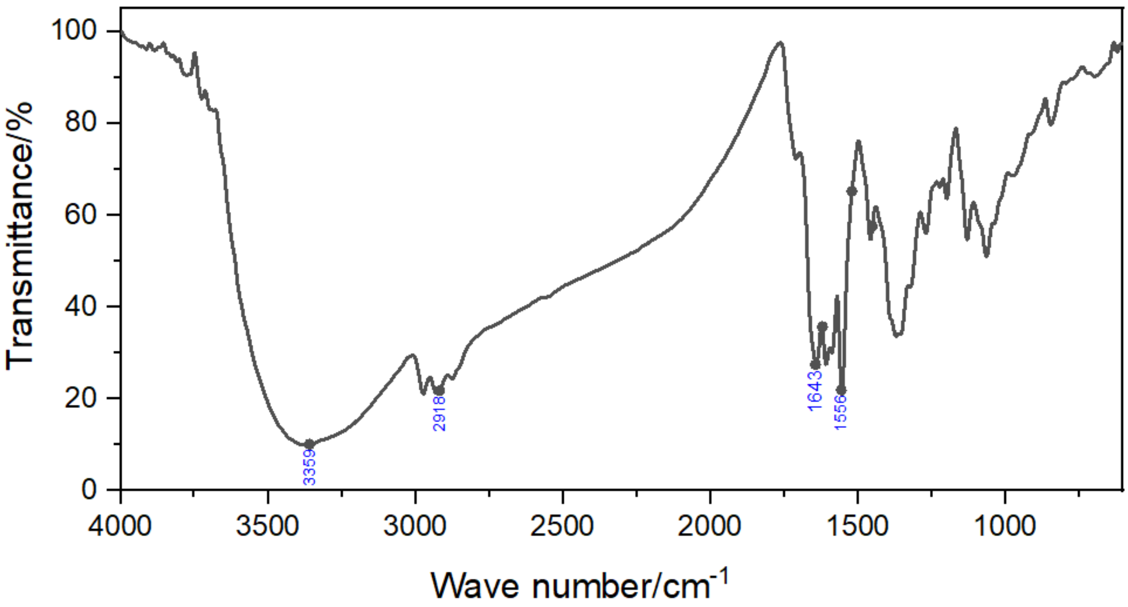

Figure S19. The IR spectrum of compound **2**

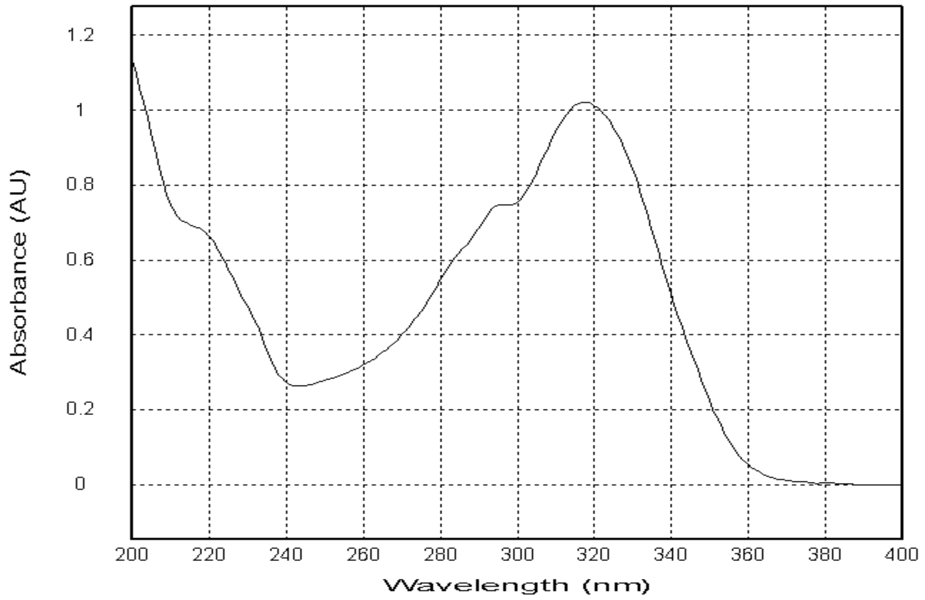

Figure S20. The UV spectrum of compound **2**

Table S1. Energy Analysis for the Conformers of (10*S*, 2′*R*) **–1.**

compound	Conformation	G (Hartree)	G (Kcal/mol)	ΔG (Kcal/mol)	Boltzmann Dist (%)
(10*S*, 2′*R*) **–1**	**1-1**	-977.0539	-613110.5756	0	46.54
(10*S*, 2′*R*) **–1**	**1-2**	-977.0526	-613109.7812	0.794425733	12.16
(10*S*, 2′*R*) **–1**	**1-3**	-977.0539	-613110.5049	0.070736633	41.30

Figure S21. B3LYP/6-311g** optimized low-energy conformers of (10*S*, 2′*R*) **–1.**

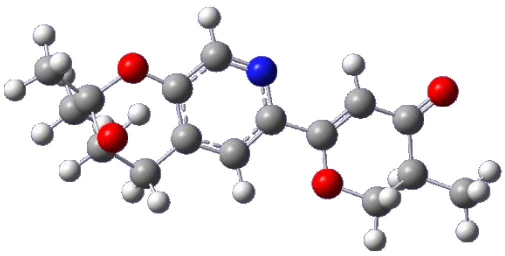

1-1 (46.54%)

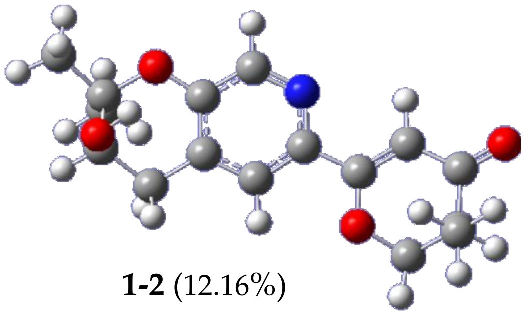

1-2 (12.16%)

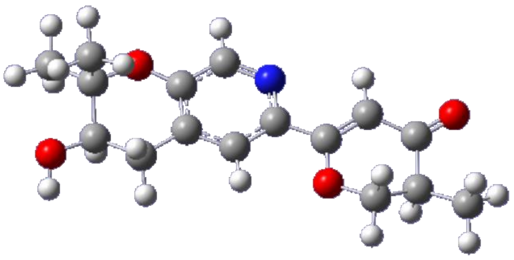

1-3 (41.30%)

Table S2. Energy Analysis for the Conformers of (10*R*, 2′*R*) **–2.**

compound	Conformation	G (Hartree)	G (Kcal/mol)	ΔG (Kcal/mol)	Boltzmann Dist (%)
(10*R*, 2′*R*) **–2**	**2-1**	-977.0539	-613110.5465	0	44.77
(10*R*, 2′*R*) **–2**	**2-2**	-977.0526	-613109.7815	0.765012481	12.30
(10*R*, 2′*R*) **–2**	**2-3**	-977.0538	-613110.5216	0.024826784	42.93

Figure S22. B3LYP/6-311g** optimized low-energy conformers of (10*R*, 2′*R*) **–2.**

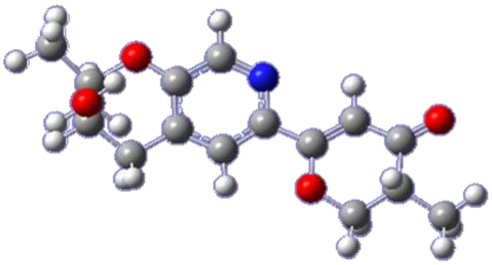

2-1 (44.77%)

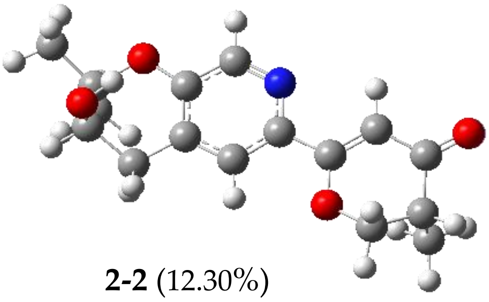

2-2 (12.30%)

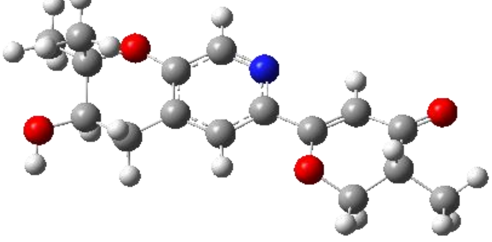

2-3 (42.93%)

Figure S23. Key NOE (purple dash arrow) correlations of compounds **1** and **2**

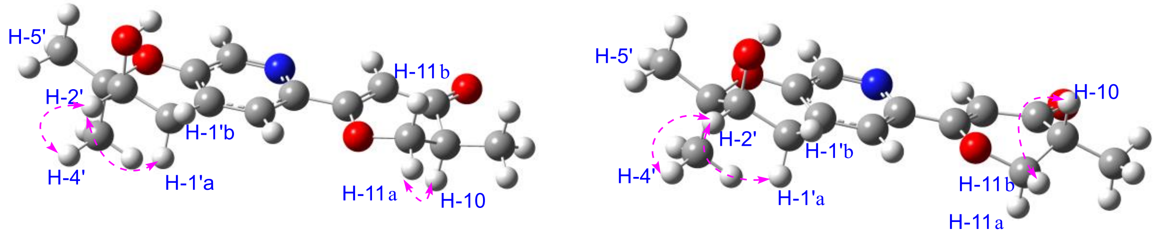

1 **2**

Figure S24. The X-ray Single crystal structure of amphichoterpenoid B (**5**) and its ECD spectra **[1]**

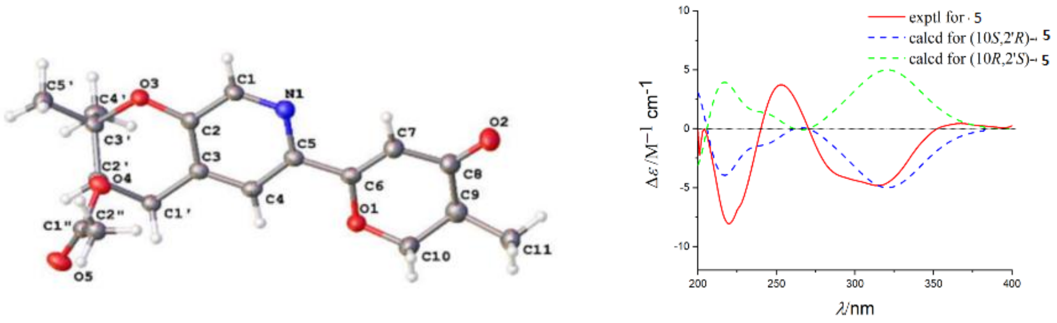

Table S3. Inhibitory activity of compounds **1–5** on AChE

Acetylcholinesterase Inhibitory Assays.

The acetylcholinesterase (AChE) inhibitory assays was carried out by modified Ellman's method [2]. Briefly, 10 μL different concentration of the tested compounds, 10 μL enzyme solution (AchE, from Electrophorus electricus, product number: C3389-2KU, Sigma-Aldrich, USA) were added to 160 μL PBS buffer (0.1M, pH 7.6) and pre-incubated for 5 min at 37 °C. Then, added 10 μL chromogenic agent, 5,5′-Dithiobis-(2-nitrobenzoic acid) (DTNB, 1.8 mg/mL) and 10 μL substrate (acetylthiocholine iodide, 1.3 mg/ml), the reaction mixture was then incubated for 12 min at 37 °C. The hydrolytic activity of the acetylthiocholine was assessed by the formation of yellow-colored 5-thio-2-nitrobenzoate anions at 410 nm. The activity was determined their absorbance at 410 nm using a 96-well microplate reader. Rivastigmine was used as a positive control. The inhibition rate of AChE was calculated by the formula [(A_0 - A_1/A_0] × 100, where A_0 is the absorbance of the blank, and A_1 is the absorbance of the compounds. All experiments were performed in triplicate, and IC_{50} values were calculated graphically according to the logarithmic concentration inhibition curves (Graphpad Prism 7.0).

Compounds [1]	IC_{50} (μM)

amphichoterpenoid D (**1**)	12.5±0.5
amphichoterpenoid E (**2**)	11.6±0.4
(+)-amphichoterpenoid A (**3**) [1]	18.8±1.2
(-)-amphichoterpenoid A (**4**) [1]	53.2±1.9
amphichoterpenoid B (**5**) [1]	23.9±0.7
amphichoterpenoid C [1]	25.7±0.6
Rivastigmine [b]	3.9±0.2

[a] IC_{50} values are represented as means ± standard deviation from three independent experiments; [b] positive control

References

[1] Jiang, M.; Wu, Z.; Wu, Q.; *et al*. *Chin Chem Lett* **2021**, *32*, 1893-1896.

[2] Ellman, G., Courtney, K., Andres J., *et al*. *Biochem. Pharmacol.*, 1961, 7, 88-95.
